# Energy Dense, Protein Restricted Diet Increases Adiposity and Perturbs Metabolism in Young, Genetically Lean Pigs

**DOI:** 10.1371/journal.pone.0072320

**Published:** 2013-08-26

**Authors:** Kimberly D. Fisher, Tracy L. Scheffler, Steven C. Kasten, Brad M. Reinholt, Gregory R. van Eyk, Jeffery Escobar, Jason M. Scheffler, David E. Gerrard

**Affiliations:** Department of Animal and Poultry Sciences, Virginia Tech University, Blacksburg, Virginia, United States of America; INRA, France

## Abstract

Animal models of obesity and metabolic dysregulation during growth (or childhood) are lacking. Our objective was to increase adiposity and induce metabolic syndrome in young, genetically lean pigs. Pre-pubertal female pigs, age 35 d, were fed a high-energy diet (HED; n = 12), containing 15% tallow, 35% refined sugars and 9.1–12.9% crude protein, or a control corn-based diet (n = 11) with 12.2–19.2% crude protein for 16 wk. Initially, HED pigs self-regulated energy intake similar to controls, but by wk 5, consumed more (*P*<0.001) energy per kg body weight. At wk 15, pigs were subjected to an oral glucose tolerance test (OGTT); blood glucose increased (*P*<0.05) in control pigs and returned to baseline levels within 60 min. HED pigs were hyperglycemic at time 0, and blood glucose did not return to baseline (*P = *0.01), even 4 h post-challenge. During OGTT, glucose area under the curve (AUC) was higher and insulin AUC was lower in HED pigs compared to controls (*P* = 0.001). Chronic HED intake increased *(P*<0.05) subcutaneous, intramuscular, and perirenal fat deposition, and induced hyperglycemia, hypoinsulinemia, and low-density lipoprotein hypercholesterolemia. A subset of HED pigs (n = 7) was transitioned back to a control diet for an additional six weeks. These pigs were subjected to an additional OGTT at 22 wk. Glucose AUC and insulin AUC did not improve, supporting that dietary intervention was not sufficient to recover glucose tolerance or insulin production. These data suggest a HED may be used to increase adiposity and disrupt glucose homeostasis in young, growing pigs.

## Introduction

The increasing prevalence of obesity represents a major public health concern. Perhaps most alarming, the frequency of childhood obesity has tripled since 1980 [1]. Obesity itself is not considered a disease; however, increased adiposity is strongly associated with aberrant metabolic and endocrine function. Altered metabolism contributes to central adiposity, dyslipidemia, hypercholesteremia, and insulin resistance [2], [3]. These factors, which constitute the metabolic syndrome, augment risk for chronic diseases. In accord, the incidence of type 2 diabetes mellitus, which was formerly considering “adult onset”, is also increasing in children [1].

Animal models of obesity and metabolic dysregulation during growth (or childhood) are limited. Pigs may be particularly useful models for understanding obesity and insulin resistance in children because humans and pigs are similar with regard to whole body size and metabolism, cardiovascular system, and digestive system [4]. Moreover, pig childhood, defined as the interval between weaning and puberty, lasts approximately 22 wk. Thus, a pig model allows ample time for dietary approaches and intervention strategies, as well as provides abundant tissue for analyses or repetitive sampling.

Dietary and genetic approaches are often incorporated into various animal models to increase body adiposity and induce metabolic dysregulation. Genetic selection for high and low subcutaneous fat depth over several generations results in lean and obese pig lines [5]. Additionally, feral Ossabaw pigs are genetically obese and deposit significantly more subcutaneous fat [4]. Yet, these changes do not appear sufficient to perturb whole body glucose homeostasis during the “childhood” period. While obese and lean lines exhibit differences in body composition at 9 to 10 wk of age [6], the obese line was not hyperglycemic, hypertryglyceridemic, or hyperinsulinemic from birth to 5.5 m [7]. Meanwhile, in comparison to lean contemporary counterparts, young Ossabaw pigs (1–6 m) are only mildly resistant to insulin action [8]. This suggests dramatic differences in genetics alone are not adequate to induce obesity-related metabolic abnormalities during the childhood period.

Altering diet composition and increasing caloric intake are additional means of inducing obesity and insulin resistance, although their effectiveness may be age and breed dependent. Adult Ossabaw pigs fed a high-fat and high-calorie diet develop characteristics of metabolic syndrome, including insulin resistance, glucose intolerance, dyslipidemia, and hypertension [2], [3], [9]–[12]. Similarly, young Göttingen minipigs, particularly females, possess increased body fatness, glucose intolerance, and insulin resistance after consuming a high energy diet for 4 months [13]. In contrast, Yucatan mini-pigs overfed a western-type diet during development and puberty are heavier at sexual maturity but do not possess increased blood glucose, indicating these pigs may have adapted to possess mechanisms for coping with excess intake [14].

Although increasing body adiposity is certainly a major risk factor for metabolic syndrome, lean mass is also an important consideration. Skeletal muscle represents a large proportion of total body mass and is the primary site for insulin-mediated glucose uptake. Thus, inherent changes in metabolic capacity or growth potential of skeletal muscle alter an individual’s susceptibility to obesity and insulin resistance. For instance, fetal pigs from the selected obese line and Ossabaw breed possess muscle characteristics, such as lower muscle DNA content [15], [16], that suggest their tendency for obesity may be related to a limited potential for muscle growth determined before birth. In contrast, contemporary pig breeds utilized for meat production have a high potential for lean growth. Because skeletal muscle represents a large proportion of total body mass, genetically lean pigs may be more resilient to diet-induced obesity. Thus, our objective was to increase adiposity and disrupt glucose metabolism in genetically lean pigs during the interval between weaning and puberty. We selected young, commercially bred female pigs and fed a low-protein, high-fat, and high-sugar diet for 16 wk. Glucose metabolism, insulin response, and hepatobiliary disorder were also evaluated to determine chronic effects of high energy diet (HED) consumption as well as to test the efficacy of nutritional intervention.

## Materials and Methods

### Ethics Statement

The Virginia Tech Institutional Animal Care and Use Committee approved all experimental procedures; this study was conducted in accordance with the Federation of Animal Science Societies’ Guide for the Care and Use of Agricultural Animals in Research and Teaching.

### Animals and Housing

Female pigs (Landrace/Large White dam × Duroc sire; Premium Genetics 1020, Murphy-Brown, Waverly, VA).obtained from a commercial swine farm were weaned at 21 d of age and transported to Virginia Tech. Pigs were individually housed in 0.6×0.9 m double-deck pens as previously described [17], [18] and offered a basal diet (Table 1) which met or exceeded nutrient recommendations [19]. Pigs were given ad libitum access to food and water, unless otherwise indicated. At 35 d of age, pigs were weighed and randomly assigned to either control (CON) or HED treatments (CON: 12.88±0.57; HED: 13.45±0.55 kg BW, *P = *0.45); trial commencement was defined as wk 0. At wk 3, pigs were moved to 1.22×1.22 m pens for the remainder of the study.

**Table 1:** Formulation and estimated composition of control (CON) and high energy (HED) diets for pigs.

	Diet							
	Basal^1^	Phase 1		Phase 2		Phase 3		Phase 4^2^
	6–9 kg	10–29 kg		30–59 kg		60–89 kg		≥90 kg
Ingredient		CON	HED	CON	HED	CON	HED	CON
Corn, g/kg	694.4	698.5	243.1	782.0	307.1	830.2	350.2	884.0
SBM, g/kg	240.0	240.0	230.0	195.0	170.0	150.0	130.0	100.0
Soy Oil, g/kg	10.0	10.0	–	–	–	–	–	–
Fishmeal, g/kg	30.0	30.0	–	–	–	–	–	–
L-lysine⋅HCl, g/kg	1.0	1.0	–	1.2	–	0.8	–	0.8
Monocalcium phosphate, g/kg	6.5	6.5	9.7	7.5	8.5	5.7	7.0	0.7
Limestone, g/kg	8.8	8.5	11.7	10.0	10.0	9.5	9.0	10.7
Vitamin premix, g/kg	0.8	1.2	1.2	0.8	0.8	0.8	0.8	0.8
Mineral premix, g/kg	0.5	0.8	0.8	0.5	0.6	0.5	0.5	0.5
Salt, g/kg	3.0	3.5	3.5	3.0	3.0	2.5	2.5	2.5
Antibiotic, g/kg	5.0	–	–	–	–	–	–	–
Beef tallow, g/kg	–	–	150.0	–	150.0	–	150.0	–
Sucrose, g/kg	–	–	200.0	–	200.0	–	200.0	–
D-fructose, g/kg	–	–	150.0	–	150.0	–	150.0	–
**Composition**								
ME^3^, kcal/kg	3375	3389	3991	3338	4007	3349	4020	3364
Crude protein, %	19.1	19.2	12.9	15.9	10.6	14.1	9.1	12.2
Fat, %	4.7	4.7	16.3	3.6	16.4	3.7	16.5	3.8
Ca, %	0.7	0.7	0.7	0.6	0.6	0.5	0.5	0.5
Available P, %	0.3	0.3	0.3	0.2	0.2	0.2	0.2	0.1

1 Diet given during the acclimation period prior to the beginning of dietary treatments.

2 Only CON Phase 4 diet was fed. Diet phase was based on average body weight within treatment. Intervention pigs consumed CON Phase 4 diet for 6 wk.

3 **Metabolizable energy**.

### Diets and Experimental Procedures

Control (n = 11) and HED (n = 12) diets were formulated and fed in phases based on nutritional requirements of pigs. Diet phase was determined according to mean body weight (BW) within treatment (Table 1). Food disappearance, BW, and ultrasound measures were collected weekly. CON and HED diets were fed for 16 consecutive wk. A sub-group of HED pigs was selected for a 6 wk dietary intervention (INT, n = 7) feeding the CON diet to simulate recovery from chronic HED consumption. At the end of respective treatment periods, pigs were euthanized by electrical stunning and exsanguination. *Longissimus* muscle samples were collected immediately following exsanguination; liver samples were collected following evisceration. Samples for biochemical analysis were snap frozen in liquid nitrogen and stored at −80°C. Halved carcasses were chilled at 4°C for 24 h. The cross-sectional area of the *longissimus dorsi* muscle (LMA) and the subcutaneous fat thickness was determined by cutting between the 10^th^ and 11^th^ costae [20]. Perirenal fat was manually dissected and weighed.

### Ultrasound

Depths of subcutaneous (USubQ) fat and *Longissimus* muscle at the last rib were collected weekly using a portable real-time ultrasound scanner (Aloka SSD-500v, Aloka Co., LTD., Wallingford, CT) with a 7.5 MHz (wk 1 to 7) or a 5 MHz (wk 8 onward) transducer (all from Aloka Co., LTD., Wallingford, CT). Vegetable oil was used as the ultrasound medium. Ultrasonic measurements were used to estimate changes in fat and lean content in individual pigs throughout treatment.

### Oral Glucose Tolerance Test (OGTT) and Clinical Characteristics

Pigs were subjected to an OGTT at wk 15 (CON and HED) and 22 (INT) of study. Pigs were fasted 12 h then offered an amount of CON diet equal to 1% BW mixed with a 40% D-glucose solution for an offering of 2 g glucose per kg BW. Animals were physically restrained and jugular venipuncture blood samples were collected in lithium-heparinized vacutainers (BD, Franklin Lakes, NJ) 0.5 h before and at 0.5, 1, 2, 3, and 4 h post offering. Blood samples were immediately analyzed for glucose (YSI 2300 STAT Plus, YSI Inc., Yellow Springs, OH), then centrifuged (3,000×g, 15 min, 4°C). Resulting plasma was collected and frozen at −80°C until analysis. Plasma insulin (Porcine ELISA, ALPCO Diagnostics, Salem, NH), low density lipoprotein (LDL), high density lipoprotein (HDL), triglyceride (TG), aspartate aminotransferase (AST), alanine aminotransferase (ALT), bilirubin, γ-glutamyl transferase (GGT), and alkaline phosphatase (ALK) were determined using commercially available kits (all from Teco Diagnostics, Anaheim, CA) according to manufacturer instructions. Insulin resistance was estimated using homeostatic model assessment (HOMA-IR) and quantitative insulin sensitivity check index (QUICKI) as previously described [21], [22]. Pancreatic β-cell function was estimated using HOMA-B [22] with the formula [fasting insulin (mU/L) × 20] ÷ [fasting glucose (mmol/L) − 2.0], which accounts for lower fasting glucose levels in pigs compared to humans.

### Proximate Analysis

Moisture and extractable lipid content of *Longissimus* muscle and liver samples were determined according to Novakofski et al. [23] with modifications. Briefly, 2 g of frozen muscle was ground, weighed, and enclosed in dried filter paper. After freeze-drying to determine moisture content, lipid was extracted in a Soxhlet apparatus using chloroform:methanol (87∶13) for 12 h. Samples were dried and weighed to determine extractable lipid content.

### Statistics

Pig was considered the experimental unit. Data normality was tested using the univariate procedure of SAS (Ver. 9.1.3, SAS Institute, Cary, NC); all data were normally distributed. The mixed procedure of SAS with repeated measures using time (week) and treatments as fixed effects and pig, pen type, and room as random effects for randomized complete-block design [24] was used to test effect of diet on tissue adiposity and plasma metabolites. BW was used as a covariate for compositional parameters; glucose intake during OGTT was used as a covariate for plasma glucose and insulin analyses. Glucose and insulin total area under the curve (AUC) during OGTT were calculated using the trapezoidal method. Data are presented as least squared means ± pooled SE. Statistical significance was determined as *P*<0.05; tendency for statistical significance was determined as *P*<0.10.

## Results

### Growth and Carcass Characteristics

**Figure 1:**
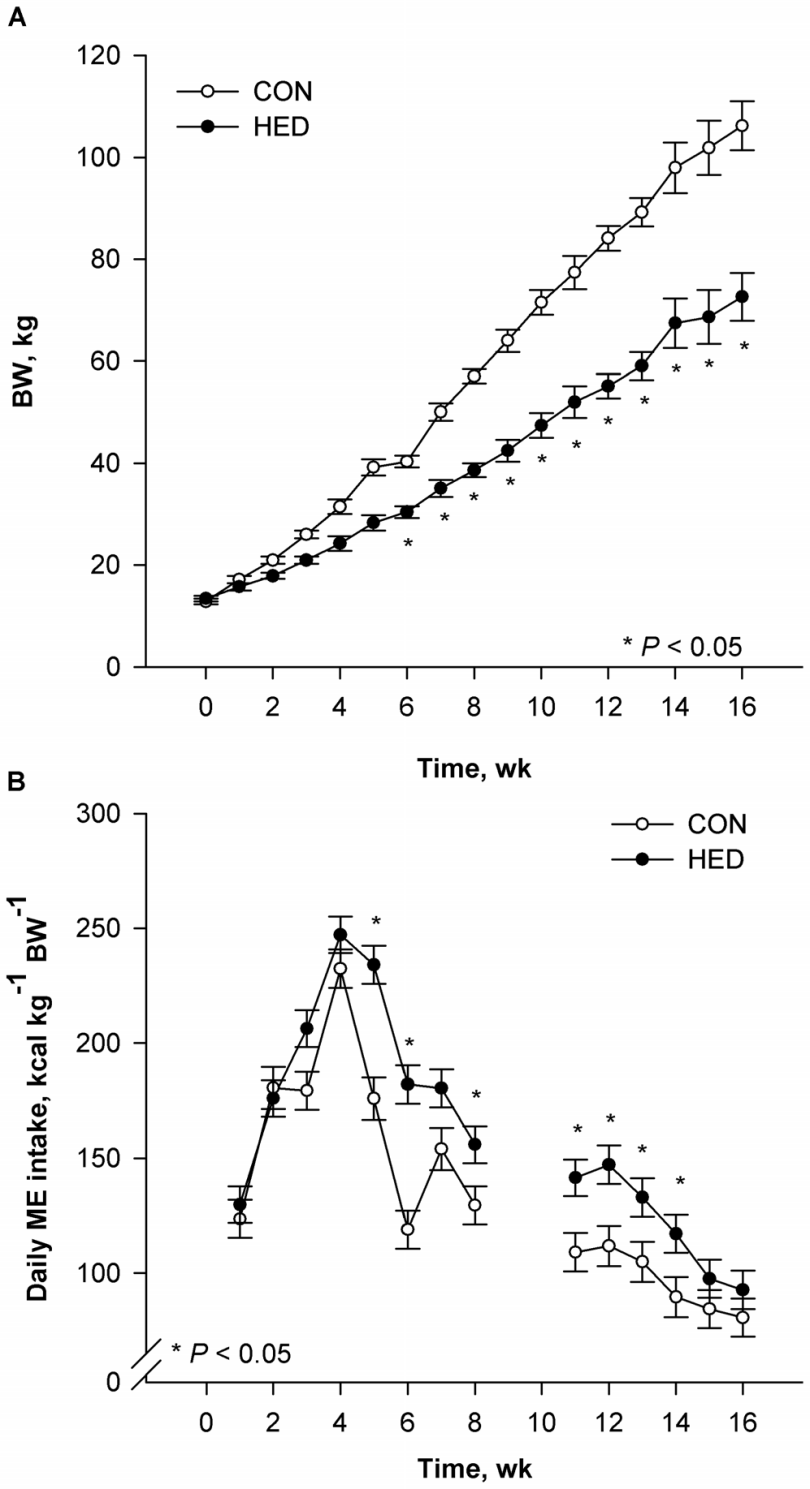
Changes in BW (A) and metabolizable energy intake (B) during a 16 wk dietary treatment. Pigs were fed control (CON, n = 11) or high energy diets (HED; n = 12) containing 15% fat and 35% refined sugars for 16 weeks. Data are presented as LS means ± SE; asterisks indicate significant differences at *P*<0.05.

Ratio of metabolizable energy (ME):standardized ileal digestible amino acid (AA) was modified in HED to alter nutrient partitioning to divert more energy toward lipid accretion. Average growth rate of HED pigs was 31% lower (*P*<0.001) than CON (Figure 1A), in agreement with our ME:AA reduction. Because of the increased energy density, HED pigs consumed less (*P*<0.0001) food daily (kg/d) than controls (data not shown). Initially, HED self-regulated relative energy intake (ME/kg BW) similarly to CON (*P* = 0.83), but, by wk 5, HED pigs consumed more (*P*<0.001) energy per kg body weight (Figure 1B). Over treatment duration, HED pigs consumed 20.6% more (*P*<0.001) calories per kg BW than CON pigs (Figure 1B inset) although data from weeks 9 and 10 were omitted due to feeder issues associated with the challenge of delivering feed with very different flow characteristics.

**Figure 2:**
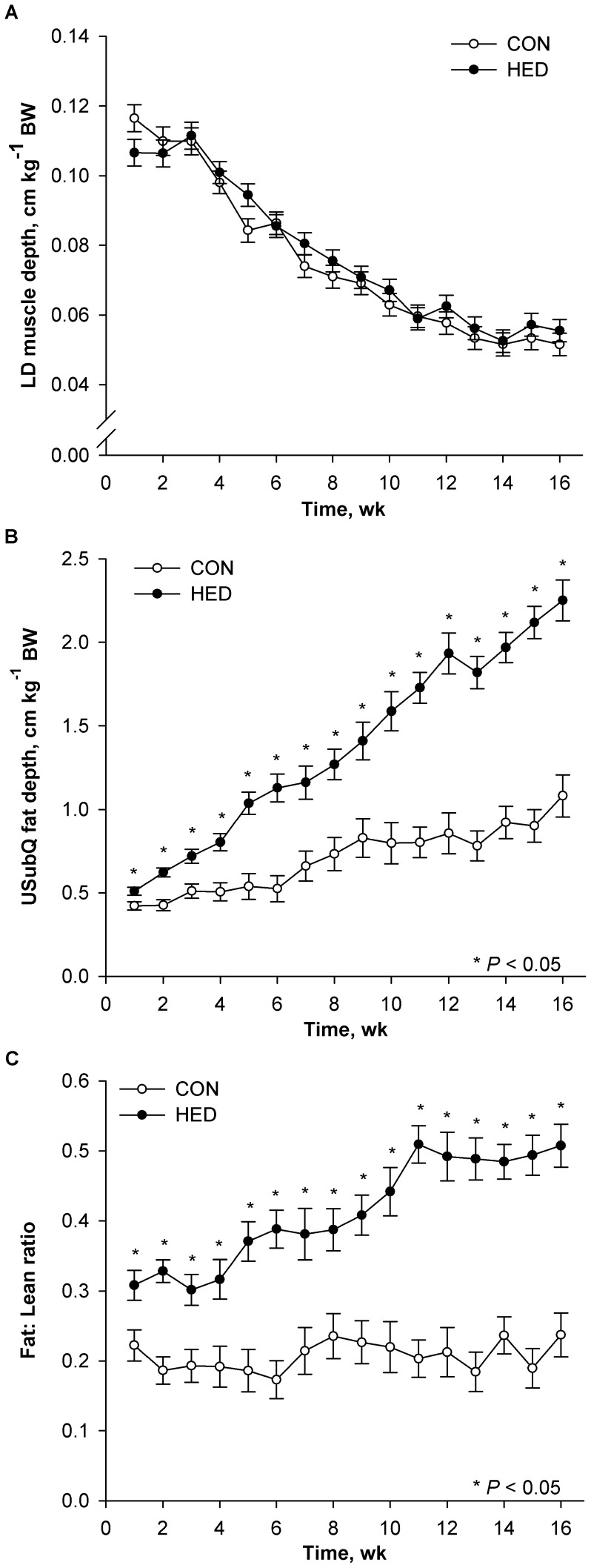
Growth traits of pigs fed control (CON) or high-energy diet (HED). Pigs were fed control (CON, n = 11) or high energy diets (HED; n = 12) containing 15% fat and 35% refined sugars for 16 weeks Adjusted ultrasonic *Longissimus dorsi* (LD) muscle depth (A), adjusted ultrasonic subcutaneous (USubQ) fat depth (B), USubQ fat deposition adjusted by LD depth (C). USubQ fat and LD depth are adjusted by BW. Data are presented as LS means ± SE; asterisks indicate significant differences from control within week at *P*<0.05.

Ultrasonic subcutaneous (USubQ) fat depth and muscle depth were determined over treatment duration to provide an index of fat and muscle compositional changes. Proportional muscle growth, depth of *Longissimus* muscle per kg of BW, was not different between treatments (Figure 2A). However, proportional fat deposition, USubQ fat depth at the last rib per unit of BW, was increased (*P*<0.05) for HED pigs (Figure 2B). Moreover, by wk 6, HED pigs deposited twice as much (*P*<0.001) USubQ fat per unit of lean compared to CON pigs (Figure 2C).

Final body weight and empty BW of CON were greater (*P*<0.01) than HED pigs (Table 2). In HED, LMA was reduced (*P*<0.001) compared to CON. Carcass SubQ fat at the 10^th^ rib was 87% thicker (*P*<0.05) in HED than CON. A 21% reduction (*P*<0.01) in SubQ carcass fat was measured in INT compared to HED pigs. Perirenal fat was increased in HED compared to Control (*P*<0.0061), and in INT compared to both CON and HED pigs.

**Table 2:** Effect of chronic dietary treatment on carcass characteristics^1^.

	Treatment				
Characteristic	Control	HED^2^	INT^3^	SE^4^	*P*-value
Body weight, kg	108.0^a^	76.0^b^	99.0^a^	5.3	0.001
Subcutaneous fat depth at the 10^th^ rib^5^, cm	1.5^a^	2.9^c^	2.3^b^	0.12	0.001
*Longissimus* muscle area^5^, cm^2^	43.2^a^	33.2^b^	31.8^b^	1.3	0.001
Perirenal fat, g per kg body weight	10.0^ a^	17.5^b^	36.2^c^	1.2	0.0001
Body fat, %^6^	23.42^a^	28.71^b^	25.49^a^	0.90	0.0016
*Longissimus* muscle Proximate moist, %	73.3^a^	70.4^b^	73.7^a^	0.54	0.01
*Longissimus* muscle Proximate fat, %	2.91^a^	8.91^b^	5.06^c^	1.43	0.001
Liver Proximate moist, %	72.9^x^	73.7^y^	72.4^z^	0.20	0.01
Liver Proximate fat, %	7.62	7.70	8.73	0.91	0.39

1 Data are least-square means per treatment, CON, n = 11; HED, n = 5; INT, n = 7.

2 High-energy diet fed over a 16 wk period.

3 Intervention at the end of HED. Pigs were transitioned to a control diet for a 6-wk period.

4 Pooled SE of treatment groups.

5 Data are body weight-corrected.

6 Calculated percent fat on carcass. [26], [27].

a,b,c Means in a row without a common superscript differ, *P*<0.05.

x,y,z Means in a row without a common superscript differ, *P*<0.10.

Proximate moisture content was higher (*P*<0.001) in *Longissimus* muscle of CON compared to HED pigs (Table 2). Fat content of *Longissimus* muscle was higher (*P*<0.05) in HED compared to controls. With dietary intervention, fat content of *Longissimus* muscle was reduced by 40% in INT compared to HED pigs. Liver moisture and fat content were not different among treatments. Carcass fat content was greater in HED pigs than controls (P<0.001), while those HED pigs switched back to a control diet (INT) showed a reduction (P<0.05) in carcass fat.

### Oral Glucose Tolerance Test

**Figure 3:**
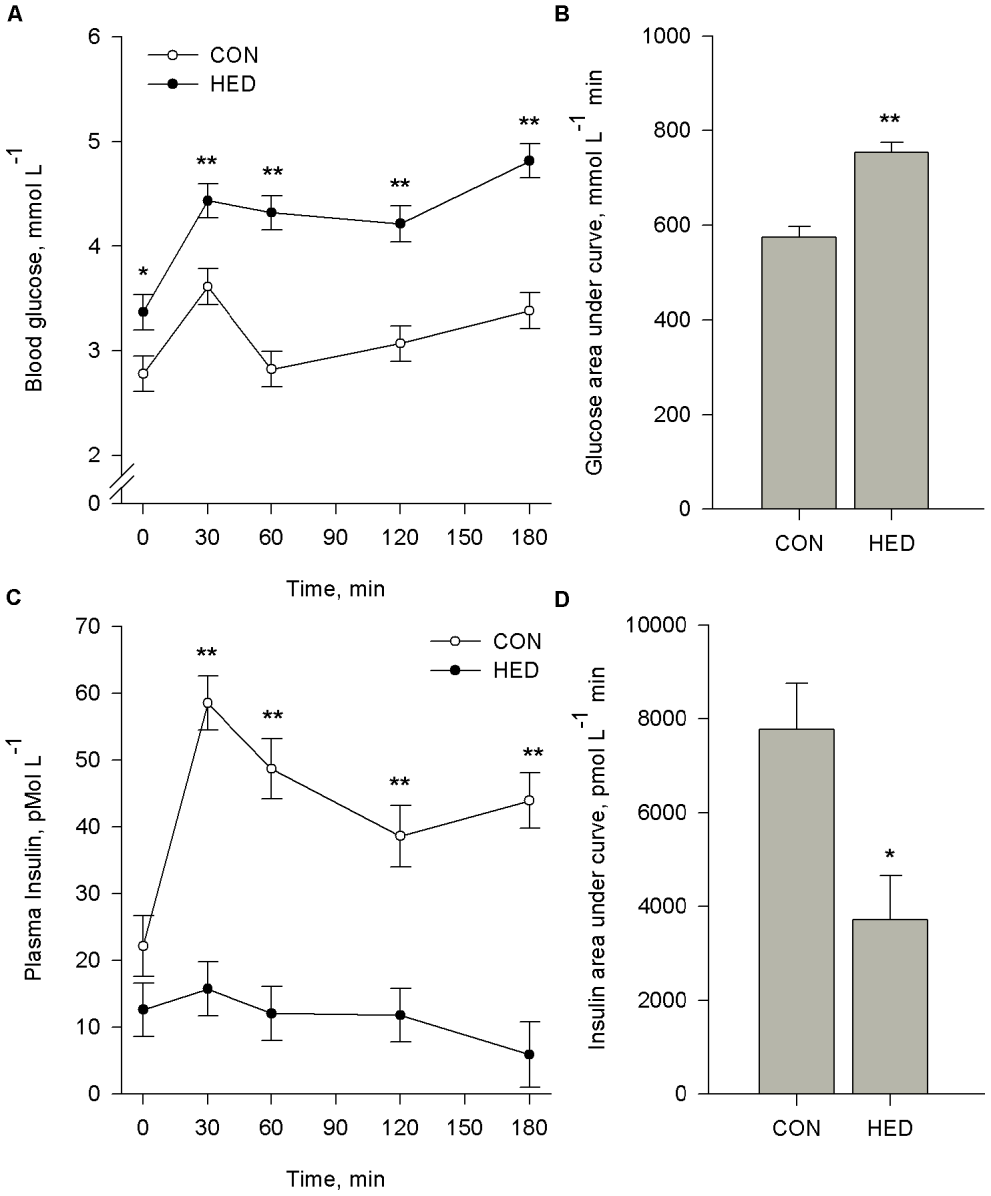
Chronic feeding of high energy diet (HED) impairs glucose clearance. Pigs fed control (CON, n = 11) or high energy diets (HED; n = 12) containing 15% fat and 35% refined sugars for 16 weeks and subjected to an oral glucose tolerance test (OGTT). Pigs were fasted overnight and subsequently challenged with an oral bolus of 2 g glucose/kg BW. Blood samples were collected at indicated time intervals to determine plasma glucose (A) and insulin (C). The respective area under curves (B,D) were calculated. Data reported are LS mean ± SEM. *means differ at P<0.05. **means differ at P<0.001.

**Figure 4:**
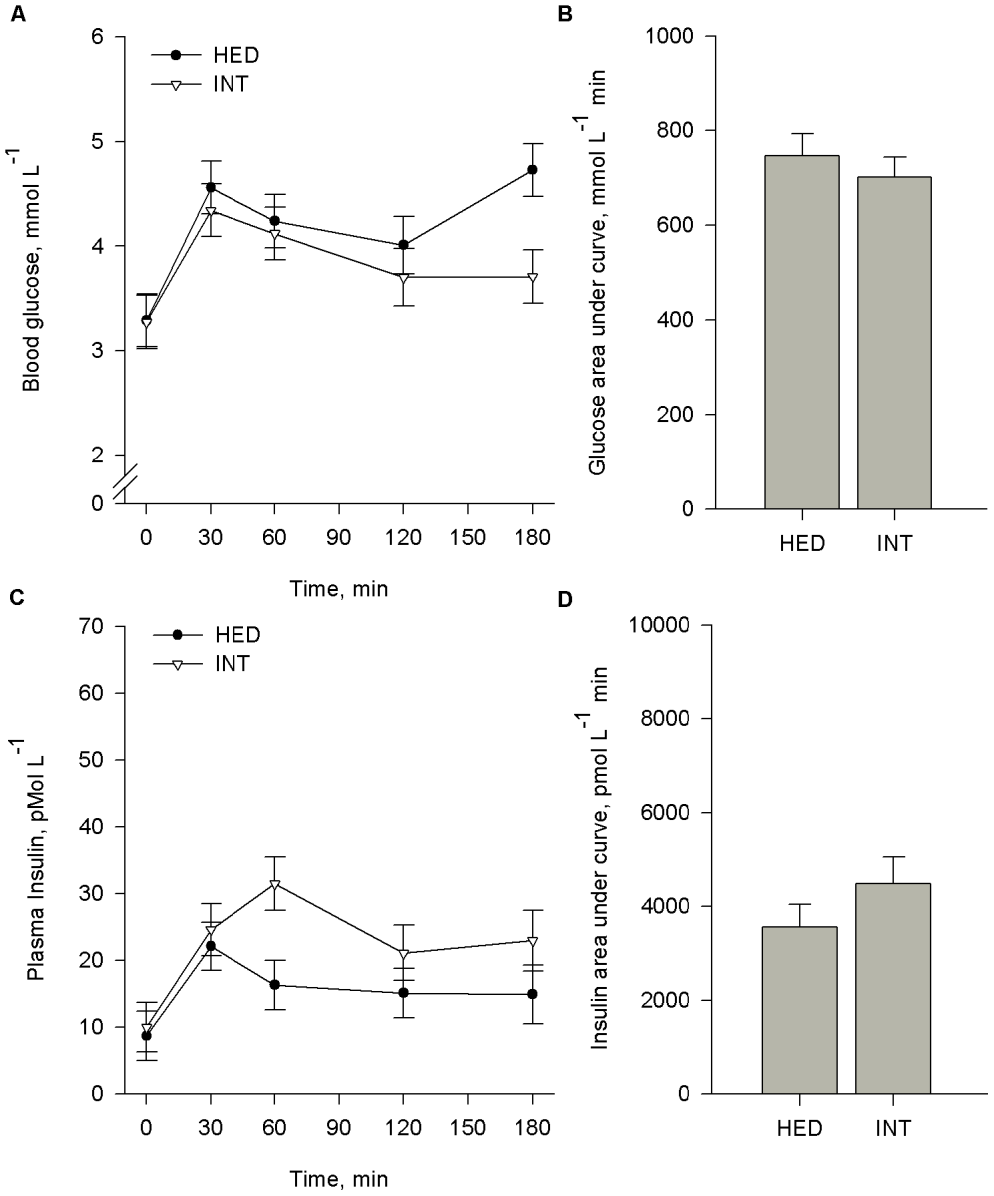
Dietary intervention after chronic high energy diet (HED) feeding does not improve glucose clearance. Pigs (n = 7) were fed diets containing 15% fat and 35% refined sugars for 16 weeks (HED) and subsequently returned to a control diet (% fat) for 6 additional weeks (INT). At the end of each feeding period, pigs were fasted overnight and subsequently challenged with an oral bolus of 2 g glucose/kg BW. Blood samples were collected at indicated time intervals to determine plasma glucose (A) and insulin (C). Comparisons were made only within pigs tested at both time points. The respective area under curves (B, D) were calculated. Data reported are LS mean ± SEM. *means differ at P<0.05. **means differ at P<0.001.

Pigs were subjected to an OGTT at wk 15 (CON and HED) and 22 (INT). Baseline blood glucose was 21% higher (*P*<0.05) in HED compared to CON pigs (Figure 3A). In response to offering a glucose bolus, a parallel increase (*P*<0.05) in blood glucose was observed from baseline to 30 min in CON, HED, and INT pigs. CON blood glucose returned to baseline levels by 60 min (*P*>0.05), whereas blood glucose from HED and INT pigs remained elevated (*P*<0.05) beyond 3 h after the sugar bolus The glucose AUC was significantly increased (*P*<0.004) for HED (Figure 3B) and maintained in INT pigs (Figure 4B).

CON pigs exhibited a more than two-fold increase (*P*<0.001) in plasma insulin within 30 min of receiving the glucose bolus (Figure 4A). In contrast, HED and INT pigs failed to mount an insulin response to the glucose bolus. The AUC for plasma insulin for HED was 52% lower (*P*<0.005) compared to controls (Figure 3D). In agreement with glucose response, dietary intervention did not result in improvement in insulin AUC (Figure 4D).

### Clinical Characteristics

At wk 16, fasting glucose was 21% higher (Figure 3) and fasting insulin was 43% lower in HED compared to CON pigs (*P*<0.001). Consequently, HOMA-IR and HOMA-B were lower whereas QUICKI was higher (*P*<0.001) in HED pigs compared to CON (Table 3). A 6 wk intervention period did not alter fasting glucose, insulin, HOMA-IR, HOMA-B, or QUICKI (Figure 4, Table 3).

**Table 3:** Effect of chronic high energy diet (HED) and subsequent intervention on plasma metabolites.

Effect of diet^1^				
	Treatment			
Characteristic	Control	HED	SEM	*P*-value
n	11	12		
QUICKI	0.510	0.562	0.012	0.005
HOMA-IR	0.243	0.155	0.019	0.004
HOMA-B	47.4	16.0	4.6	0.0001
LDL-cholesterol, mmol/L	75.0	101.8	3.46	0.0001
HDL-cholesterol, mmol/L	48.2	54.2	2.0	0.043
Triglycerides, mmol/L	59.2	67.8	3.3	0.073
ALT^2^, U/L	24.2	21.7	1.9	0.34
ALK^3^, U/L	13.3	26.9	1.9	0.0001
AST^4^, U/L	25.5	24.3	2.2	0.69
GGT^5^, U/L	16.0	22.1	1.3	0.003
Bilirubin, mg/L	17.9	32.4	4.7	0.037
**Effect of intervention** 6				
	**Treatment**			
**Characteristic**	**HED**	**INT**	**SEM**	***P*** **-value**
n	7	7		
QUICKI	0.551	0.565	0.014	0.52
HOMA-IR	0.165	0.154	0.013	0.58
HOMA-B	18.6	18.9	3.4	0.94
LDL-cholesterol, mmol/L	94.1	76.56	4.2	0.012
HDL-cholesterol, mmol/L	54.3	50.5	3.1	0.41
Triglycerides, mmol/L	64.9	58.3	3.8	0.25
ALT^2^, U/L	19.3	33.8	3.0	0.002
ALK^3^, U/L	27.6	15.9	3.0	0.016
AST^4^, U/L	25.6	22.5	2.2	0.33
GGT^5^, U/L	23.3	25.5	2.4	0.52
Bilirubin, mg/L	28.1	15.0	4.1	0.043

1 HED was fed to pigs for 16 wks starting at 5 wks of age.

2 Alanine aminotransferase.

3 Alkaline phosphatase.

4 Aspartate aminotransferase.

5 γ-Glutamyl transferase.

6 At the end of the 16 wk treatment, a subset (n = 7) of HED pigs were transitioned back to the control diet for an additional 6 wk. LSmeans comparing only the subset pigs at the beginning (HED) and end of the intervention period (INT) are reported.

Fasting LDL levels at wk 16 were 35.7% higher (*P*<0.003) in HED compared to CON, and dietary intervention reduced (*P*<0.01) fasting LDL concentrations by 18% following the 6 wk dietary intervention. Similarly, plasma TG levels tended to be higher (*P*<0.07) in HED compared to CON pigs. Dietary intervention did not influence plasma TG levels.

Plasma ALT was elevated (*P*<0.002) in intervention pigs compared to HED pigs at wk 16 (Table 3). Plasma GGT was higher (*P*<0.03) in HED and was not altered by dietary intervention. Plasma ALK and bilirubin levels were higher (*P*<0.05) in HED relative to CON pigs, but then declined (P<0.05) during the intervention to levels similar to CON. AST levels did not different among treatments.

## Discussion

Obesity is a health concern primarily because it increases the risk for type 2 diabetes and other chronic diseases. Central obesity, along with insulin resistance, impaired glucose tolerance, dyslipidemia, and hypertension, are a cluster of risk factors; presence of at least three of these criteria constitutes metabolic syndrome [2], [3], [9], [25]. To this end, our objective was to induce obesity and metabolic dysregulation in pre-pubertal pigs. Because current pig models for obesity may be predisposed to reduced muscle growth potential and/or altered muscle metabolism, we chose to use commercially bred, genetically lean pigs that possess high potential for lean growth.

Pigs fed the HED exhibited greater adiposity, evidenced by increased subcutaneous fat and perirenal fat. Based on prediction equations for fat-free lean [26] and bone [27], pigs fed the HED exhibited an estimated 23% increase in body fat. This is a conservative estimate; body fat of HED pigs is likely underestimated because the equation relies primarily on subcutaneous fat, whereas perirenal and mesenteric depots are not included. While increased deposition of fat in adipose tissue is noteworthy, accumulation of lipid in muscle, liver, and pancreas may be more significant harbingers of metabolic disease [28]. Intramuscular triglyceride content is increased with obesity and is a strong predictor of insulin resistance in humans [29]. Pigs fed the HED deposited roughly three times more intramuscular fat. Although we suspected liver steatosis in HED pigs, the fat content was similar among treatments. In contrast to humans and rodents, in which the primary site for de novo lipogenesis is liver [30], the primary site in pigs is adipose tissue [31]. Moreover, pigs have a low ketogenic capacity and unique regulation of hepatic fatty acid oxidation [32]. Together, this likely explains why ectopic fat deposition did not occur in livers of HED pigs and is consistent with data reported for Ossabaws fed high fat and sucrose diets [33]. Alternatively, inclusion of increased levels of cholesterol or sodium cholate may be necessary to induce development of fatty liver [11]. We also examined activity of liver enzymes in plasma as markers of hepatocellular health. Both ALK and GGT activity, as well as bilirubin content, were elevated in HED pigs. Increased activity of liver enzymes is associated with obesity and liver pathology [34], [35], suggesting HED negatively impacted liver health.

Additionally, chronic feeding of HED elicited development of other markers of metabolic dysregulation. Pigs fed the HED exhibited elevated plasma LDL and tended to possess higher triglyceride levels, although this increase is likely not pathological and less dramatic than that observed in Ossabaw pigs fed high fat, high cholesterol diets [2], [11], [36]. More importantly, HED pigs demonstrated impaired glucose tolerance and insulin response to glucose challenge, as well as signs of reduced pancreatic beta cell function. Thus, our experimental diet induces obesity and metabolic dysregulation in young, growing pigs. It is unclear if the profound lack of insulin response to glucose challenge is due to impaired ability of the pancreas to sense glucose, produce insulin, release insulin, or a combination; and to what extent the pancreas functionality may be compromised. Iberian sows fed a high fat diet and sampled by a similar method also failed to mount an insulin response to glucose challenge [37]. By contrast, adult Göttingen minipigs fed a high fat diet for a shorter period (three months) showed elevated fasting insulin and insulin response to glucose challenge [38] which may be attributed to either duration of treatment or genetics. Stress is known to cause an acute reduction in β-cell function [39] which would be elevated by repeated restraint for blood draws and likely explains the general rise in blood glucose without further increase in insulin after 30 min. Production of various gut peptides involved in regulating insulin secretion [40], [41] may be altered by dietary treatment and would be relevant in an oral glucose challenge. Further investigation is necessary to determine factors that contribute to impaired insulin response and the extent to which pancreas function can be restored.

We readily acknowledge our high energy diet was intentionally deficient in protein and this reduces lean body growth and repartitions dietary nutrients toward fat deposition [42]–[45]. In addition to reduced growth rate and lean deposition, there may be other metabolic effects that could be attributed to protein restriction rather than increased energy density and consumption of refined sugars. However, reports of these other effects are limited, and generally associated with much more extreme protein restriction [46], [47] Many obesigenic or atherogenic diets routinely fed to mini-pig and Ossabaw models are also deficient in protein. These dietary shortcomings are created by simply adding fat, sugar or other high energy ingredients to regular pig chow [2], [9]–[11], [36], [48]–[54], which dilutes the protein and amino acid component below the effective lean growth needs of the animal. This manipulation of the metabolizable energy to amino acid ratio dramatically alters the composition of weight gain in growing pigs [2], [9]–[11], [42], [48]–[52], [55], [56]. Even so, animals fed high energy, low protein diets grow and accrete lean tissue, albeit in different proportions and rates compared to pigs fed diets designed to maximize lean gain. In agreement, HED pigs grew slower and weighed less than control pigs at the end of the study. Because this approach causes a reduced whole body growth, we cannot rule out the possibility that high energy, low protein diet alters endocrine status or the nutritional physiology, which drives adiposity. Clearly, this is quite different from what occurs with most obese children. Not only do obese children grow faster on a weight basis, they tend to be taller than contemporaries, up to age 14 years [57]. Moreover, a diet deficient in protein rarely occurs in developed countries. Even though HED pigs grew slower and were smaller, when tissue weights were adjusted for body weight, muscle growth of HED pigs was similar to controls, but fat deposition was undoubtedly greater. This disparity in growth-related composition was further exacerbated when the major indicator of the fatness, subcutaneous fat, was adjusted for the major indicator of muscularity, the depth of the longissimus muscle (Figure 2C).

Initially, HED pigs consumed a similar amount of energy as control pigs. Indeed, pigs have the ability to self-regulate energy intake based on metabolic needs [43]–[45]. However, by 5 wk, HED pigs consumed more energy per unit body weight. This suggests that after considerable dietary energy assault, regulatory mechanisms controlling intake fail, resulting in caloric consumption in excess of requirements. This strongly argues the existence of a biological mechanism capable of gauging dietary energy intake in juvenile pigs. Components of this hypothetical whole-body energy-sensing mechanism are not known but deserve some attention. For example, leptin and insulin act on the hypothalamus to regulate energy balance and hepatic gluconeogenesis, and therefore, appear to control fasting hyperglycemia in diabetes [58], [59]. As such, low levels of circulating insulin in our pigs potentially mean low levels of hypothalamic insulin signaling; this may contribute to fasting hyperglycemia and dysregulated energy intake. Certainly, defining the mechanisms that regulate energy intake is important for understanding how dietary factors contribute to obesity and metabolic dysregulation.

The underlying cause of childhood obesity is thought to result from an imbalance between total calories consumed versus calories expended [60]; however, others suggest the type of calorie consumed is as important as the total calories [61]–[63]. Pigs fed isonitrogenous, isocaloric diets containing either low or high fat exhibit differences in adipose deposition. Remarkably, a high fat diet increases carcass adipose regardless of genetic tendency for leanness or obesity, supporting that dietary fat is preferentially utilized for adipose deposition [64]. Similarly, the macronutrient composition of the diet influences body composition during weight loss. Obese rats that are restriction fed a high carbohydrate diet exhibit decreased body fat percent and enhanced insulin sensitivity compared to rats fed an isoenergetic low carbohydrate diet [65]. Moreover, carbohydrates differ vastly in their glycemic index, or glucose-raising potential. Refined grain and sugar products typically possess higher glycemic indices compared to whole grains and unprocessed vegetables [66]. Long-term consumption of high glycemic index diets may contribute to increased energy intake and altered body composition, as well as physiological and hormonal changes that promote insulin resistance [67]. The typical Western diet derives about 36% of energy from refined sugars and oils [66]. Whereas typical commercial pig diets contain less than 5% fat and no refined sugar, our HED contained 15% fat and 35% refined sugar. Thus, macronutrient composition and relatively high glycemic index may be significant factors contributing to increased adiposity and glucose intolerance in our HED pigs.

In an attempt to reverse diet-induced metabolic syndrome, a subset of HED pigs was exposed to 6 wk dietary intervention. Curiously, feeding a low-fat control diet for 6 wk did not improve glucose tolerance or insulin production. However, improvement in other measures of metabolic stress was observed, as evidenced by LDL-cholesterol, TG, ALK, and bilirubin levels returning to normal. Thus, a longer period of dietary intervention may be necessary to improve glucose and insulin homeostasis. Alternatively, it is possible HED irreversibly damages pancreatic β-cell function, and “normal” insulin production cannot be fully recovered. Thus, more drastic interventions, such as dietary energy restriction and exercise, may be necessary to promote insulin-independent glucose uptake and improve whole-body energy metabolism [68].

In summary, chronic feeding of a high energy, low protein diet to pre-pubertal pigs results in increased adiposity. More importantly, these pigs exhibit insulin resistance, LDL hypercholesterolemia, and hepatobiliary disorder. Altogether, this demonstrates that dietary manipulation induces metabolic dysregulation in young, growing pigs, and corroborates its utility as a potential model for childhood obesity.
